# Cold-related symptoms and performance degradation among Thai poultry industry workers with reference to vulnerable groups: a cross-sectional study

**DOI:** 10.1186/s12889-020-09272-6

**Published:** 2020-09-04

**Authors:** Nipaporn Auttanate, Chotirot Chotiphan, Suchinda Jarupat Maruo, Simo Näyhä, Kirsi Jussila, Sirkka Rissanen, Penpatra Sripaiboonkij, Tiina M. Ikäheimo, Jouni J. K. Jaakkola, Wantanee Phanprasit

**Affiliations:** 1 Department of Occupational Health, Faculty of Public Health, Mahidol University, Bangkok, Thailand; 2 Center for Environmental and Respiratory Health Research, University of Oulu, P.O. Box 5000, FI-90014 Oulu, Finland; 3 Finnish Institute of Occupational Health, Työterveyslaitos, FI-90032 Helsinki, Finland; 4 School of Public Health, Physiotherapy and Sports Science, University College Dublin, Belfield, Dublin, DB4, Ireland

**Keywords:** Poultry industry, Cold, Occupation, Symptoms

## Abstract

**Background:**

Few studies have examined cold-related symptoms among cold workplace workers in Thailand. This study aimed to determine the prevalence of cold-related cardiorespiratory, circulatory, and general symptoms and performance degradation among Thai chicken industry workers and identify vulnerable groups.

**Methods:**

Overall, 422 workers aged from 18 to 57 years at four chicken meat factories in Thailand were interviewed for cold-related symptoms and complaints. The results were expressed in terms of model-based adjusted prevalence and prevalence differences (PDs) in percentage points (pp) with 95% confidence intervals (CIs).

**Results:**

In total, 76.1% of the respondents reported cold-related respiratory symptoms, 24.6% reported cardiac symptoms, 68.6% reported circulatory symptoms, and 72.1% reported general symptoms. In addition, 82.7% of the respondents reported performance degradation. Cold-related respiratory symptoms increased by PD 29.0 pp. (95% CI 23.4–34.6) from the lowest to the highest educational group, with a similar pattern observed in performance degradation. Forklift drivers and storage and manufacturing workers complained of cold-related respiratory symptoms more than office staff (PD 22.1 pp., 95% CI 12.8–31.3; 12.0 pp., 95% CI 2.4–21.6; and 17.5 pp., 95% CI 11.5–23.6, respectively); they also reported more performance degradation (PD 24.1 pp., 95% CI 17.0–31.2; 19.8 pp., 95% CI 14.1–25.6; and 14.8 pp., 95% CI 8.0–22.6, respectively). Weekly alcohol consumers reported more performance problems owing to cold (PD 18.2 pp., 95% CI 13.9–22.6) than non-consumers of alcohol. Cardiac and circulation symptoms were more common in women than men (PD 10.0 pp., 95% CI 1.1–18.9; and 8.4 pp., 95% CI 0.5–16.4, respectively). The age trend in performance issues was curved, with the highest prevalence among those aged 35–44 years, while the oldest workers (45–57 years) perceived less cold-related symptoms, particularly thirst.

**Conclusions:**

Cold-related symptoms and performance degradation were found to be common in this industry, with vulnerable groups comprising of highly educated workers, forklift drivers, storage and manufacturing workers, weekly alcohol consumers, aging workers, and women. The results demonstrate a need for further research on the adequacy of protection provided against the cold, particularly given that global warming will increase the contrast between cold workplaces and outdoor heat.

## Background

In Thailand, the poultry industry is one of the largest sectors of the food industry, which is notorious for having a high risk for occupational diseases [1]. Hygienic requirements presuppose working at low temperatures, ranging from approximately 0 °C to 15 °C in production halls to − 20 °C in cold storages [2]. Low temperatures combined with physical loading factors may precipitate respiratory [3], cardiovascular [4], and musculoskeletal symptoms, as well as peripheral circulation disturbances, particularly in fingers and hands [5–9]. Low temperatures also negatively affect physical and mental performance [10, 11] and lead to decreased work ability and productivity.

Both short- and long-term exposure to cold air causes inflammatory changes in the airways and worsening of respiratory function and may precipitate asthma attacks in predisposed individuals [3]. During normal winter in a northern climate, 25–29% of people experience shortness of breath, wheezing, or prolonged coughing, which are attributed to the cold [10]. Information on such symptoms in artificially cooled environments and specific occupational groups is limited, but in Thailand, most workers in the frozen food processing industry complain of respiratory symptoms at work [12]. Decreased lung function has been reported among cold storage workers [13]. However, people living in cold climates have adapted to low temperatures and are less sensitive to occupational cold exposure [7].

Exposure to cold causes cutaneous vasoconstriction, increase in blood pressure, and consequently, increase in the cardiac load. This condition is further aggravated through physical exercise at work and may manifest as cardiac symptoms such as chest pain or arrhythmias. Other consequences of cold exposure include haemoconcentration, which increases the risk of vascular thrombosis and myocardial infarction, and arrhythmias, which may ensue through a reflectory mechanism [14]. In Finland, it has been reported that the cold causes cardiac symptoms in approximately 5% of the general population and in 5–6% of those working in outdoor occupations [15]. Cold-related disturbances in the peripheral circulation are also common: one-third of workers in the Thai frozen food industry [12] and 20% in the freezing coffee industry [16] complain of such symptoms, compared with a lower prevalence (12–15%) reported for the general population in a northern climate [10]. The effects of cold-related cardiac and respiratory complaints are not limited to symptoms because they can increase future mortality and hospital admissions by a factor of two [17].

Information on the prevalence of health complaints among cold workplace workers in Thailand is limited to one study involving seven symptom groups [12]; however, this study did not address specific symptoms of diseases or cold-related performance degradation, and considered a limited number of confounding factors only. To provide more information on the prevalence of cold-related symptoms and complaints, we conducted a cross-sectional study among Thai chicken industry workers in four factories, where the mean temperatures in various departments and job categories ranged from approximately − 20 °C to 23 °C. We asked about 22 individual cold-related symptoms, including performance degradation, and we allowed for relevant personal and work-related factors. In particular, we aimed to identify the subgroups especially vulnerable to workplace cold in relation to sex, age, education, body weight, smoking status, alcohol consumption, job category, and employment years.

## Methods

### Population

Overall, 422 workers in four chicken meat factories belonging to the same company were interviewed by trained interviewers. The factories are located in the central and north-eastern parts of Thailand, where outdoor temperatures at the time of the study (July to November 2017) ranged from 28 °C to 34 °C (Additional file 1: Table S1). The work consists of chicken meat cutting, processing, and packing. Manufacturing workers cut and shape chicken meat and package it. Forklift drivers transport the packages from production halls to cold storages and shipping yards. Storage workers move and lift the packages in the cold storage areas and transfer the packages from the shipping yards to trucks. Office workers normally deal with administrative duties in offices. For most workers, protective clothing is provided by the employer. The clothing is specifically intended for use in protecting the workers from the cold but has no quality certification. The manufacturing workers wear a coverall, a hair net, a balaclava, a plastic apron, rubber gloves, and boots. The storage workers wear a thick overcoat, a balaclava or beanie, cotton gloves, sneakers, or shoes. The forklift drivers have an insulated hooded coverall, cotton gloves, and safety shoes. The office workers wear long trousers. All the workers have a lunch break of one hour in the workplace canteen. Medical checkups are offered to the workers once a year.

The number of participants needed was determined according to Hsieh et al. [18] using the formula: n = (Z_1-α/2_ + Z_1- β_)^2^/[P (1-P) β*^2^], where Z_1-α/2_ was 1.96, and power was 0.90 (Z_1- β_ = 1.28). The calculations were performed based on one group vs. reference group comparisons, but multi-class comparisons were undertaken in final regression analyses. We assumed an outcome prevalence of 50% and an effect size of 0.3 standardised units (β*), which corresponds to a small difference. The calculations indicated that a sample size of 420 would be sufficient to detect such a prevalence difference with a probability of 0.90. Considering the variety of symptoms asked, we expected to see widely varying prevalence figures. Thus, in cases of 10% or 20% prevalence, for example, the sample would be sufficient to detect differences of β* = 0.5 and β* = 0.4 standardised units (medium-size effects), respectively, with the same probability.

Approximate quotas for the number of workers to be interviewed in each factory were set in advance, and the final number of participants was determined in relation to their availability during regular working hours and the work schedules of the study team. All workers at the selected factories (288, 5054, 500, and 7250 people across the four factories, respectively) were invited to participate by a local study supervisor. A worker first gave his/her consent to participate, after which another worker was sought to replace him/her at the worksite, and permission to participate was obtained from the supervisor. During the lunchbreaks, the workers had rest and could not be interviewed. Altogether, 422 workers (59, 145, 70, and 148 across the four factories, respectively) were available and interviewed during their working time. Additional file 1: Table S1 provides summary information on the number of workers and temperatures among the base population and among the sample population.

Air temperature, relative humidity, and air velocity were measured in the working spaces of approximately 300 participants in this study (a subsample of all the 422 interviewed workers) working in cold storage and production halls as well as in offices and other factory sections whose workers were willing to participate (Additional file 1: Table S1). This subsample was used as secondary data to describe workplace physical conditions but was not used in the main data analysis (Additional file 1: Table S1 and Table S2). The main difference between the larger interviewed sample and the subsample measured in terms of these variables was that the subsample involved fewer individuals working in office areas.

### Questionnaire

The first part of the questionnaire inquired into details concerning personal characteristics, living habits, and work-related factors (Table 1). In the second part, cold-related symptoms and complaints were elicited through the following questions “Have you experienced any of the following symptoms during work or after work because of the cold?” and “Does the cold decrease your performance at work?” There were 22 individual symptoms or complaints, some of which were merged to form larger categories (respiratory, cardiac, circulatory, finger, and general symptoms) from responses to the first question, and a performance degradation category from responses to the second question, with 16 symptoms or symptom categories for assessment, as shown in Table 2. Other questions were asked concerning what temperature the participant regarded as cold, how long during the day the participant worked at temperatures < 0 °C and 0–16 °C, and how often the participant moved between cold and warm working spaces. The questions were derived from an international standard [20] and were modified for the present purpose based on experiences from previous cold studies [10, 15, 21–23].

**Table 1:** Characteristics of the sample

Characteristic	Coding	No. (%)
Sex	Males	197 (46.7)
	Females	225 (53.3)
Age (yr)	18–24	100 (23.8)
	25–34	136 (32.3)
	35–44	121 (28.7)
	45–57	64 (15.2)
Education	Primary school	69 (16.6)
	Middle school	97 (23.3)
	High school	72 (17.3)
	Vocational school	72 (17.3)
	University	106 (25.5)
Body mass index (kg/m^2^)^a^	Normal (≤ 22.9)	192 (45.6)
	Overweight (23.0–24.9)	68 (16.2)
	Obese (≥ 25.0)	161 (38.2)
Smoking	Never smoked	306 (72.5)
	Ex-smoker	18 (4.3)
	Regular smoker	98 (23.2)
Alcohol consumption	Does not consume alcohol	206 (49.2)
	Occasionally	102 (24.3)
	Monthly	63 (15.0)
	Weekly	48 (11.5)
Factory section	Cold storage	166 (39.6)
	Production room	152 (36.3)
	Office	95 (22.7)
	Other	6 (1.4)
Job category	Forklift driver	33 (8.2)
	Storage worker	95 (23.5)
	Manufacturing worker	142 (35.1)
	Office staff	134 (33.2)
Employment years	0–1.9	139 (33.2)
	2–9.9	138 (32.9)
	10 +	142 (33.9)
Working hours/day	0–9.9	197 (47.1)
	10+	221 (52.9)
Physical strain at work	Light sitting	141 (33.5)
	Other light	98 (23.3)
	Medium heavy	111 (26.4)
	Heavy	71 (16.9)
All		422

^a^ Classified according to World Health Organization [19]

One participant had missing information on age, one for body mass index, one for work strain, three for alcohol consumption, three for factory section, three for employment years, four for working hours and six for educational class, and for 18 participants information on job category was missing or remained indeterminate

**Table 2:** Prevalence (%) of cold-related symptoms and complaints during work or after work

Symptom/complaint	Both sexes (*N* = 422)	Male (*N* = 197)	Female (*N* = 225)	Female - male difference
Respiratory^a^	76.1	78.7	73.8	−4.9
Cardiac^b^	24.6	27.2	22.3	−4,9
Circulation^c^	68.6	71.4	66.2	−5.2
Fingers^d^	61.8	65.0	58.9	−6.1
General complaints	72.1	73.6	70.9	−2.7
Sleep disturbances or intermittent sleep	21.2	15.3	26.3	11.0
Unusually strong fatigue	43.0	42.6	43.3	0.7
Thirst	41.0	39.1	42.7	3.6
Dryness of mouth	57.0	57.7	56.5	−1.2
Performance degradation	82.7	79.2	85.8	6.6
Concentration	46.4	35.0	56.4	21.4
Motivation	42.3	32.0	51.3	19.3
Endurance	55.0	48.2	60.9	12.7
Ability to hold	36.9	31.0	42.2	11.2
Hand grip force	71.7	68.0	75.0	7.0
Finger dexterity	71.0	67.5	74.1	6.6

^a^ Shortness of breath, mucus excretion, prolonged cough or wheezing

^b^ Chest pain or cardiac arrhythmia

^c^ Peripheral circulation disturbances, blurring of vision or migraine

^d^ Cold sensitive, white or blue fingers

### Data analysis

The presence of each symptom and complaint was coded as binary and used as response variate in generalized linear regression with logit link function and quasibinomial error distribution. Models were run for each outcome, with the factor of interest as the explanatory factor, first adjusting for personal characteristics (sex, age, education, body mass index, smoking status, and alcohol consumption) and then additionally adjusting for selected work-related factors (factory, job category, and employment years). Other factors considered were the factory section, daily working hours, and physical strain at work, but these factors did not appreciably change the estimates of the parameters of interest (e.g., the job category), indicating no significant confounding, and were omitted from the final analyses. The analyses were conducted using the svyglm function available in R software 3.50 [24], allowing for stratified sampling with the svydesign function.

Along with the crude prevalence figures, the results from regression analysis were reported in terms of average marginal estimates for each variable of interest. The average marginal estimate involves a calculation of averages of all model-estimated proportions [25, 26]. This estimate can be seen as prevalence adjusted for all other factors in the model and was converted to a prevalence difference (PD, in percentage points, pp) from a selected reference level. Prevalence differences were used instead of relative measures, because they describe the symptom burden in this population in absolute terms and better quantify the preventive potential in each subgroup. The marginal estimates were obtained using the svypredmeans function in R.

## Results

### Sample characteristics

Of the respondents, 46.7% were men, and 84.8% were aged < 45 years (Table 1). Regarding education level, 25.5% of the respondents had university education, and 17.3% had vocational school education; of these, 76.5 and 59.4%, respectively, worked in offices. Concerning lifestyle indicators, 38.2% of the respondents were classified as obese, 23.2% were smokers, and 11.5% consumed alcohol at least once a week. Most workers were manufacturing workers (35.1%), followed by office staff (33.2%) and storage workers (23.5%), with 8.2% being forklift drivers. The respondents had been working at their current factory for an average of 7 years, 3 of which had been in cold work. The daily working time averaged 9 h, 3 of which were in the cold. Twenty-six respondents (6.2% of 422) reported elevated blood pressure diagnosed by a doctor, and < 5% reported a respiratory, joint, skin, or back condition, asthma, diabetes mellitus, or a heart condition.

Additional file 1: Table S1 and Table S2 summarise the workplace physical conditions. The mean temperature was 4 °C, ranging from − 2 °C in cold storage sections to 23 °C in other sections, ranging from − 20 °C among forklift drivers to 22 °C among the office workers, and ranging from − 2 °C among the high-school-educated workers to 11 °C among the university-educated workers. However, the worksite temperatures for individual workers varied from − 22 °C to 23 °C. The mean relative humidity was 47% and varied from 32% among the forklift drivers to 65% within office departments, while the individual values varied from 27 to 72%. The mean air velocity was 0.43 m/s, ranging from 0.30 m/s among the storage workers to 0.57 m/s among the forklift drivers, with individual variations ranging from 0 to 3.00 m/s.

Additional file 1: Table S3 summarizes the daily hours the workers in various jobs and educational categories spent at different temperatures and how often they moved between cold and warmer working sites. An average of 1.5 h per day was spent in very cold temperatures (< 0 °C), and this varied from 0.2 h/day among the office staff to 5.1 h/day among the forklift drivers. A total of 3.3 h/day on an average was spent in moderate cold temperatures (0–16 °C), ranging from 0.6 h/day among the office staff to 6.9 h/day among the manufacturing workers. A total of 77.2% of the workers (ranging from 67.0 to 87.9% in various categories) reported that they moved between cold and warmer working sites at least four times a day.

The respondents’ opinions concerning what temperature they considered to be cold are summarized in Additional file 1: Table S4. The most common temperature regarded as being cold was 20 °C, with the exception that manufacturing workers most commonly regarded 10 °C as cold. A temperature of 20 °C was regarded as cold by 47.7% of the respondents, with wide variations between subgroups, with 20 °C regarded as cold by 70.5% of the office workers and 63.5% of the university-educated workers, but only by 34.8% of the primary-school-educated workers and 16.2% of the manufacturing workers.

### Number of cold-related symptoms and complaints

The subjects reported an average of 9.6 various cold-related symptoms (Additional file 1: Table S5). The number of symptoms increased consistently according to workers’ educational level, from 8.0 symptoms among primary school educated workers to 11.2 symptoms among university educated workers. Office workers showed more symptoms (10.7) than workers in other job categories (8.9–9.4). The symptoms also increased from the age group 18–24 years to the age group 35–44 years, with some decline thereafter.

### Overall prevalence of cold-related symptoms and complaints

In total, 76.1% of the respondents reported that workplace cold caused respiratory symptoms, and 24.6% reported cardiac symptoms (Table 2). In addition, circulatory symptoms were common (68.6%), as were finger symptoms (61.8%). Furthermore, 72.1% of the respondents reported general cold-related symptoms such as strong fatigue (43.0%) or thirst (41.0%), and 82.7% reported that workplace cold worsened their performance, mostly in terms of handgrip force (71.7%) and dexterity (71.0%). In particular, worsened concentration and motivation to work were more often reported by women than by men.

### Adjusted prevalence of cold-related symptoms according to personal and work-related factors

#### Sex

Even though lower proportions of women than men reported cardiorespiratory, circulation and finger symptoms (Table 2), the adjusted prevalence of these symptoms was higher in the women (Table 3, Additional file 2: Fig. S1). In addition, despite more women reporting worse performance due to the cold (Table 2), the adjusted prevalence of these complaints was PD 11.7 pp. lower (95% CI -20.0– − 3.5) in the women (Table 4, Additional file 2: Fig. S1).

**Table 3:** Prevalence (P) of cold-related respiratory, cardiac and circulation symptoms and prevalence differences (PD, percentage points, pp)

*Subgroup*	Respiratory				Cardiac				Circulation			
	Crude		Adjusted		Crude		Adjusted		Crude		Adjusted	
	P (%)	PD (pp)	PD (pp)	95% CI	P (%)	PD (pp)	PD (pp)	95% CI	P (%)	PD (pp)	PD (pp)	95% CI
*Sex*												
Men	78.7	0.0	0.0		27.2	0.0	0.0		71.4	0.0	0.0	
Women	73.8	−4.9	1.5	(−5.7–8.7)	22.3	−4.9	10.0	(1.1–18.9)	66.2	−5.2	8.4	(0.5–16.4)
Age (yr)												
18–24	80.0	0	0.0		20.0	0.0	0.0		61.6	0.0	0	
25–34	78.7	−1.3	−4.8	(−12.5–2.9)	29.1	9.1	6.3	(−2.9–15.6)	73.5	11.9	1.8	(−7.3–11.0)
35–44	76.9	−3.1	−5.0	(−13.5–3.6)	25.0	5.0	−3.6	(− 12.1–4.9)	72.7	11.1	7.9	(−0.5–16.4)
45–57	62.5	−17.5	−9.2	(−22.4–3.9)	21.9	1.9	1.0	(−14.5–16.4)	60.9	−0.7	6.3	(−7.0–19.5)
Education												
Primary school	65.2	0.0	0.0		20.3	0.0	0.0		62.3	0.0	0.0	
Middle school	77.2	12.0	16.0	(4.3–27.7)	23.7	3.4	0.3	(−10.3–10.9)	66.7	4.4	4.7	(−6.3–15.6)
High school	69.4	4.2	7.3	(−6.0–20.5)	28.6	8.3	5.9	(−5.2–17.1)	69.4	7.1	−1.1	(−13.9–11.7)
Vocational school	81.9	16.7	24.3	(15.6–32.9)	27.8	7.5	6.3	(− 6.5–19.0)	72.2	9.9	7.6	(−4.4–19.6)
University	83.0	17.8	29.0	(23.4–34.6)	23.8	3.5	1.7	(−8.5–12.0)	71.7	9.4	8.1	(−2.1–18.2)
Body weight												
Normal	77.1	0.0	0.0		25.1	0.0	0.0		67.5	0.0	0.0	
Overweight	70.6	−6.5	−6.9	(−18.1–4.3)	20.9	−4.2	−2.4	(− 13.0–8.2)	67.6	0.1	−3.1	(−15.5–9.4)
Obese	77.6	0.5	0.0	(−6.8–6.8)	25.6	0.5	2.8	(−5.7–11.3)	70.8	3.3	4.0	(− 3.8–11.8)
Smoking												
Never	76.8	0.0	0.0		24.3	0.0	0.0		69.9	0.0	0.0	
Ex-smoker	77.8	1.0	−5.9	(−29.4–17.7)	44.4	20.1	25.2	(1.6–48.7)	72.2	2.3	−6.4	(−31.7–18.9)
Smoker	73.5	− 3.3	− 6.8	(−19.0–5.5)	21.6	−2.7	−5.7	(− 15.8–4.4)	63.9	− 6.0	− 20.6	(− 36.7– −4.4)
Alcohol												
Does not use	73.8	0.0	0.0		22.5	0.0	0.0		65.0	0.0	0	
Occasionally	75.5	1.7	−3.1	(−12.3–6.1)	23.8	1.3	−5.0	(−14.4–4.4)	71.3	6.3	7.0	(−2.7–16.7)
Monthly	79.4	5.6	−6.8	(−19.3–5.8)	23.8	1.3	−13.1	(−22.1– − 4.0)	71.4	6.4	3.4	(−9.4–16.2)
Weekly	85.4	11.6	2.7	(−10.0–15.4)	35.4	12.9	−0.1	(−13.2–13.0)	75.0	10.0	8.2	(−6.1–22.5)
Job												
Office worker	75.4	0.0	0.0		24.1	0.0	0.0		67.2	0.0	0.0	
Manufacturing	72.5	−2.9	17.5	(11.5–23.6)	26.2	2.1	−7.7	(−16.5–1.2)	67.6	0.4	3.3	(−5.8–12.4)
Storage worker	81.1	5.7	12.0	(2.4–21.6)	26.3	2.2	−8.9	(−18.1–0.4)	71.3	4.1	−2.6	(−14.6–9.4)
Forklift driver	81.8	6.4	22.1	(12.8–31.3)	18.8	−5.3	−12.4	(−26.9–2.1)	75.8	8.6	16.5	(4.1–28.8)
Employment years												
0–1.9	78.4	0.0	0.0		20.4	0.0	0.0		68.1	0.0	0.0	
2–9.9	79.0	0.6	2.4	(−5.0–9.8)	29.9	9.5	4.2	(−3.7–12.1)	72.5	4.4	1.3	(−6.7–9.4)
10–30	71.1	−7.3	0.2	(−8.0–8.4)	23.9	3.5	3.9	(− 7.6–15.3)	66.9	−1.2	− 7.1	(−18.3–4.0)

^a^ Adjusted for all variables in the table other than the variable of interest, plus factory

**Table 4:** Prevalence (P) of cold-related finger, general and performance complaints and prevalence differences (PD, percentage points, pp)

Subgroup	Finger				General				Performance degradation			
	Crude		Adjusted		Crude		Adjusted		Crude		Adjusted	
	P (%)	PD (pp)	PD (pp)	95% CI	P (%)	PD (pp)	PD (pp)	95% CI	P (%)	PD (pp)	PD (pp)	95% CI
Sex												
Men	65.0	0.0	0.0		79.2	0.0	0.0		79.2	0.0	0.0	
Women	58.9	−6.1	−0.3	(−9.7–9.1)	76.8	−2.4	−0.4	(−7.8–7.0)	85.8	6.6	−11.7	(−20.0– −3.5)
Age (yr)												
18–24	58.0	0.0	0.0		78.0	0.0	0.0		75.0	0.0	0.0	
25–34	66.2	8.2	−1.6	(−11.3–8.1)	83.8	5.8	6.7	(−0.7–14.1)	84.6	9.6	9.8	(2.1–17.6)
35–44	60.0	2.0	−1.4	(−11.6–8.8)	79.3	1.3	6.6	(−1.1–14.4)	86.8	11.8	16.9	(12.2–21.5)
45–57	62.5	4.5	0.8	(−16.2–17.8)	61.9	−16.1	−13.3	(−30.4–3.7)	82.8	7.8	16.6	(9.6–23.6)
Education												
Primary school	55.1	0.0	0.0		69.6	0.0	0.0		75.4	0.0	0.0	
Middle school	57.7	2.6	5.1	(−7.7–17.9)	78.4	8.8	−2.3	(−12.0–7.3)	74.2	−1.2	10.1	(−1.8–22.1)
High school	63.9	8.8	5.9	(−6.8–18.7)	73.6	4.0	−7.6	(−20.6–5.3)	81.9	6.5	11.2	(−1.0–23.5)
Vocational school	70.4	15.3	21.1	(10.5–31.7)	74.6	5.0	−10.7	(−25.1–3.7)	88.9	13.5	23.7	(17.0–30.3)
University	63.2	8.1	18.7	(8.0–29.4)	86.8	17.2	3.2	(−6.5–13.0)	91.5	16.1	29.3	(26.0–32.7)
Body weight												
Normal	63.4	0.0	0.0		78.6	0.0	0.0		84.4	0.0	0.0	
Overweight	60.3	−3.1	−10.9	(−24.5–2.7)	79.1	0.5	4.2	(−4.7–13.2)	77.9	−6.5	−11.9	(−23.5– −0.2)
Obese	60.9	−2.5	−4.7	(−13.6–4.1)	77.0	−1.6	−3.0	(−11.2–5.3)	82.6	−1.8	−5.8	(− 12.9–1.2)
Smoking												
Never	61.6	0.0	0.0		79.0	0.0	0.0		84.6	0.0	0.0	
Ex-smoker	61.1	−0.5	6.0	(−16.9–29.0)	77.8	−1.2	−9.0	(−37.8–19.8)	88.9	4.3	12.2	(9.0–15.4)
Smoker	62.2	0.6	−2.4	(−15.7–10.9)	74.5	−4.5	−11.3	(− 25.3–2.7)	75.5	−9.1	− 2.5	(− 12.9–7.9)
Alcohol												
Does not use	60.0	0.0	0.0		76.6	0.0	0.0		81.1	0.0	0.0	
Occasionally	63.7	3.7	10.5	(1.6–19.4)	74.5	−2.1	−4.4	(−14.2–5.4)	83.3	2.2	9.9	(2.6–17.2)
Monthly	65.1	5.1	−6.2	(−20.4–8.0)	90.5	13.9	9.9	(2.6–17.2)	82.5	1.4	7.0	(−2.5–16.5)
Weekly	64.6	4.6	−0.1	(−16.1–15.9)	77.1	0.5	−6.3	(−21.8–9.2)	91.7	10.6	18.2	(13.9–22.6)
Job												
Office worker	59.4	0.0	0.0		79.7	0.0	0.0		88.1	0.0	0.0	
Manufacturing	63.4	4.0	10.6	(0.6–20.6)	73.2	−6.5	5.3	(−4.4–15.0)	77.5	−10.6	14.8	(8.0–22.6)
Storage worker	63.2	3.8	4.2	(−8.2–16.5)	84.2	4.5	12.9	(5.3–20.5)	83.2	−4.9	19.8	(14.1–25.6)
Forklift driver	57.6	−1.8	12.4	(−4.0–28.7)	78.8	−0.9	12.8	(1.1–24.4)	87.9	−0.2	24.1	(17.0–31.2)
Employment years												
0–1.9	62.6	0.0	0.0		85.6	0.0	0.0		80.6	0.0	0.0	
2–9.9	63.8	1.2	0.2	(−8.8–9.2)	75.4	−10.2	0.0	(−7.1–7.2)	82.6	2.0	−1.6	(−7.9–4.8)
10–30	58.9	−3.7	−8.3	(−20.5–3.8)	73.0	−12.6	−5.1	(−15.6–5.4)	85.2	4.6	−8.5	(−18.1–1.1)

^a^ Adjusted for all variables in the table other than the variable of interest, plus factory

#### Age

Cold-related performance degradation increased with advancing age (Table 4, Additional file 2: Fig. S2). In particular, cold-related problems in relation to concentration, motivation, and endurance increased up to middle age (35–44 years), peaking at PD 26.5 pp. (95% CI 15.5–37.5), 32.2 pp. (95% CI 21.2–43.3), and 33.7 pp. (95% CI 25.5–42.0), respectively; however, the prevalence curve showed downward movement among workers aged 45–57 years. Especially the prevalence curve for thirst moved downward in the highest age group to PD − 32.5 pp. (95% CI -46.2– − 18.8), as did dryness of mouth (PD − 14.8 pp., 95% CI -32.9–3.2).

#### Education

**Fig. 1:**
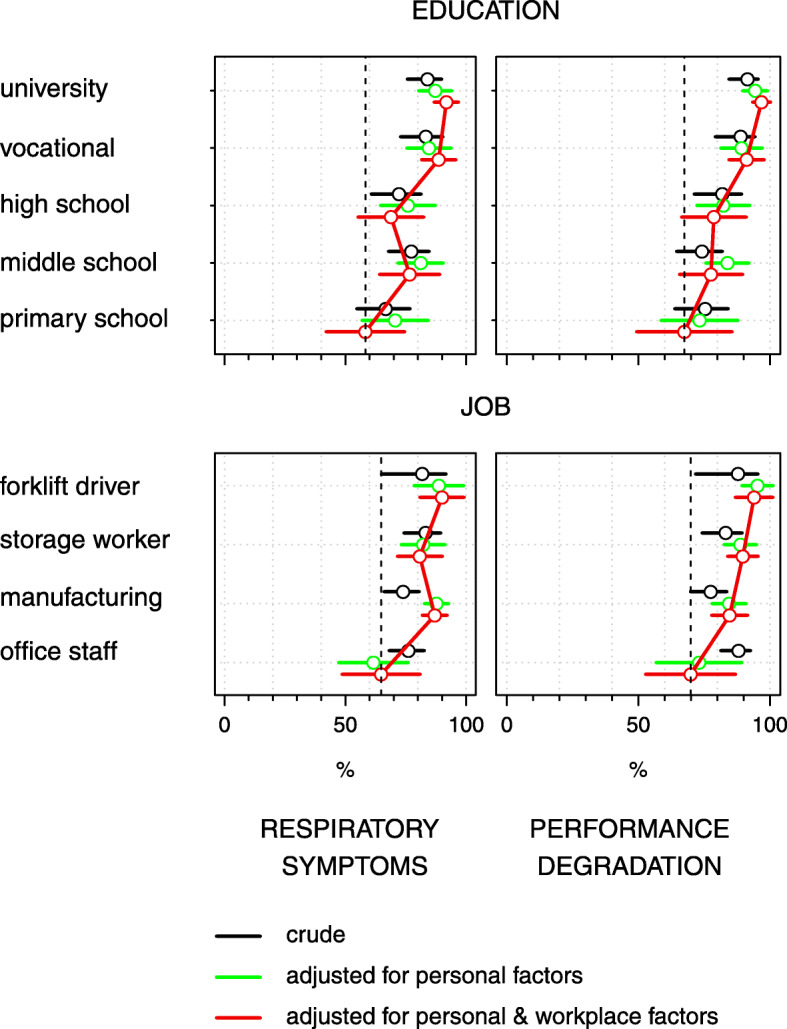
Prevalence of cold-related respiratory symptoms and performance degradation (%) by educational classes and job categories. Figure shows educational and job differences in prevalence of cold-related respiratory symptoms and performance degradation and the effect of adjusting for personal factors and additionally adjusting for work-related factors. Horizontal bars are 95% confidence intervals

The prevalence of cold-related respiratory symptoms increased by PD 29.0 pp. (95% CI 23.4–34.6) in line with increasing educational levels, from the lowest to the highest educated group (Table 3, Fig. 1), and increases of a similar magnitude were seen in finger symptoms (Table 4, Additional file 2: Fig. S3). An even more consistent increase in terms of educational level was observed in cold-related performance degradation (Table 4, Fig. 1) and separately for all individual performance symptoms (Additional file 2: Fig. S3).

#### Other personal characteristics

Body mass index was not associated with most symptoms (Tables 3 and 4), but cold-related problems with motivation (PD − 10.7 pp., 95% CI -19.5– − 1.9), endurance (PD − 8.7 pp., 95% CI -18.2–0.7), handgrip force (PD − 8.4 pp., 95% CI − 17.0–0.1) and dexterity (PD − 8.9 pp., 95% CI -17.4– − 0.5) were marginally less common among workers who were obese than normal weight workers (Additional file 2: Fig. S4). Ex-smokers had more cardiac symptoms and most types of performance issues (Table 3) than workers in other smoking categories, whereas smokers had a low prevalence of circulatory symptoms (Table 4, Additional file 2: Fig. S5). Alcohol consumers who drank on a weekly basis had more cold-related performance problems than non-consumers (PD 18.2 pp., 95% CI 13.9–22.6) (Table 4) but less thirst (PD − 24.7 pp., 95% CI -35.9– -13.5) (Additional file 2: Fig. S6).

#### Job categories

Cold-related respiratory symptoms increased across the job categories from the office workers to the manufacturing and storage workers and to the forklift drivers (Fig. 1), and some excess of circulatory symptoms was seen among the forklift drivers (PD 16.5 pp., 95% CI 4.1–28.8) (Table 3). Cold-related performance degradation similarly increased from the office workers to the forklift drivers (PD 24.1 pp., 95% CI 17.0–31.2) (Fig. 1, Table 4), as did worsening handgrip force (PD 23.0 pp., 95% CI 13.3–32.7) and dexterity (PD 17.5 pp., 95% CI 6.2–28.8) (Additional file 2: Fig. S7). In addition, general cold-related symptoms (Table 4), especially fatigue, increased in a similar fashion. Thirst and dryness of the mouth were common among the storage workers (PD 23.9 pp., 95% CI 11.2–36.5) and PD 22.1 pp. (95% CI 11.7–32.5), respectively, as well as thirst among the manufacturing workers (PD 14.1 pp., 95% CI 4.1–24.2) (Additional file 2: Fig. S7). Figure 1 and Additional file 2: Fig. S7 also show that many of these trends across the job categories, for example, in relation to respiratory and circulatory symptoms and performance degradation, were only shown using adjusted figures, with little or no differences shown in the unadjusted figures.

#### Employment years

Workers who had been employed for ≥10 years reported fewer cold-related performance problems than those with a work history of < 2 years (PD − 8.5 pp., 95% CI -18.1–1.1) (Table 4). The difference was even greater when separately considering endurance (PD − 21.3 pp., 95% CI -32.6– − 10.0) and handgrip force (PD − 15.1 pp., 95% CI -26.5– -3.7) (Additional file 2: Fig. S8).

## Discussion

### Summary of findings

In our study, chicken meat industry workers were shown to suffer a wider range of cold-related symptoms and complaints than previously recognized. We estimated the prevalence of a number of respiratory, cardiac, peripheral circulation, finger, and general symptoms and complaints and of performance degradation, as well as combinations of symptoms. The prevalence of symptoms was generally high, and furthermore, several subgroups of workers especially vulnerable to cold exposure were identified, such as highly educated workers, forklift drivers, storage and manufacturing workers, aging workers, and weekly alcohol consumers. In these subgroups, the absolute prevalence excess of at least one symptom or complaint was substantial, indicating marked gains that could be achieved by prevention. Smaller excesses limited to fewer symptom types were seen in women. Our findings add further relevant evidence-based data to that previously reported concerning the occurrence of cold-related symptoms and complaints and performance degradation among food industry workers [5, 8, 9, 12, 27] and are useful in determining effective preventive measures.

### Overall prevalence of symptoms

International guidelines have defined cold in the workplace to be temperatures < 10 °C [20]; however, in the factories concerned, 16 °C was considered sufficiently cold to presuppose the need for protective measures. Our finding of a high prevalence of cold-related symptoms and the high number of various symptoms was, therefore, not unexpected. In a previous Thai study, 41–91% of frozen food industry workers were reported to suffer from cold-related respiratory symptoms [12], while percentages of 73–82% in various job categories were observed in the present study. Figures much lower than these have been reported from northern climates. In Finland, for example, only 0–16% of meat industry workers [23] and one-fourth of the general population have been reported to complain of cold-related respiratory symptoms [10]. In this study, circulatory symptoms were reported by 67–76% of the participants in varying job categories, which was higher than the prevalence range of 18–51% reported in a previous Thai study [12] and higher than the 0–12% [23] and 12–15% [10] prevalence ranges found among Finnish meat industry workers and the Finnish general population, respectively. While 25% of the participants in this study complained of cardiac symptoms due to workplace cold, < 12% of cold workers did so in Finland [23], the prevalence of such symptoms being 4% in the Finnish general population [10]. While the effects of cold temperatures on cardiac function are well-known [4], no information is available on the prevalence of cold-induced symptoms of cardiac disease in other working populations. A previous Thai study [12] did not report cardiac symptoms separately, although 34% of the workers in that study were reported to have had cardiovascular symptoms.

Cold-related finger symptoms were reported by 62% of the participants in this study, whereas 49% of workers in Brazilian pig slaughterhouses were reported to have had finger symptoms [9], and 48 and 84% of workers in the Thai [12] and Finnish food industries [5], respectively. The cold is known to impair manual performance, especially dexterity [8, 9, 28–30], at skin temperature of 15 °C and below [31]. In our study, 71% of the workers reported cold-induced impairment of dexterity at work. Comparable prevalence figures from other working populations are difficult to obtain; however, approximately 70% of the general population in Finland have been reported to have such symptoms [10]. Cold work is also known to impair handgrip force [30, 32, 33]. In total, 72% of the participants in this study reported cold-related impairment of handgrip force, but comparable prevalence figures from other populations are not available. However, cold-related impairment of hand and finger functioning is reportedly common in cold workplace workers, despite the use of overlapping gloves [9], and may predispose these workers to accidents.

Exposure to the cold has been reported to worsen mental performance, such as concentration [34, 35]. In our sample, highly educated and middle-aged workers in particular were shown to be susceptible to concentration and motivational issues when working in the cold; however, comparable information in the literature could not be found.

### Prevalence of symptoms according to personal and work-related factors

An unexpected finding not previously reported was the association of a high level of education with cold-related respiratory and finger symptoms and worsened performance. Also the number of individual symptoms increased with increasing educational level. This finding contrasts with studies showing that higher levels of education are associated with better health [36]. However, in this occupational setting, the more highly educated staff mostly worked in office premises where the relative humidity was high (65%) and the temperature was approximately 20 °C, which 64% of the office workers considered cold. The highly educated staff and office workers also spent short times in the cold and occasionally experienced cold, and most of them (67 and 70%, respectively) moved between cold and warmer working sites at least 4 times a day, which may have caused additional thermal stress and produce cold symptoms. Repeated exposures to cold during the day may also lower body temperature [33].

Considering the lower critical temperature of approximately 22–27 °C at which heat production to maintain thermal balance starts in a lightly clothed individual [37, 38], and a recommended neutral indoor temperature of 26 °C in Thailand [39], cold-related symptoms occurring among the highly educated staff whose working temperature was 11 °C on an average were likely. Furthermore, some individual highly educated workers were exposed to below-zero temperatures (Additional file 1: Table S2), which was even more likely to have produced cold-related symptoms. Excessive cooling of office premises in a tropical climate due to effective air conditioning [40] could also lead to cold-related symptoms.

One factor affecting the highly educated staff could be their adaptation to outdoor temperatures of around 30 °C and consequent sensitivity to office temperatures several degrees lower, possibly also due to inadequate clothing. In Thailand, the effect of adaptation to the local climate and of vulnerability to temperatures below the optimal temperature is best shown by cold-related mortality, which starts at temperatures as high as 29 °C, while the threshold can be as low as 15 °C in cooler countries [41]. In addition, the most common opinion among the workers was that 20 °C is cold, suggesting a high sensitivity to the cold among this population. It is not evident why the manufacturing workers regarded a lower temperature of 10 °C as cold, but it may be that they have better protective clothing or that they have adapted to the relatively low temperature (6 °C) in the production halls.

It is possible that the more highly educated workers may be more aware of cold hazards and may tend to answer according to what they think might be expected, which may have caused bias. In any case, the excessive prevalence of cold-related symptoms among the more highly educated staff was substantial. Especially since cold-related cardiorespiratory symptoms may indicate an increased risk for actual disease events during long-term follow-up [17], intensified measures are needed to protect these workers. Appropriate measures would include regulation of office temperatures, wearing more clothing, and regulating the work-rest cycles.

The forklift drivers, whose working environment was coldest (− 20 °C), driest (relative humidity 32%), and windiest (air velocity 0.57 m/s), showed a high prevalence of cold-related respiratory symptoms, circulatory symptoms, excessive fatigue, and reduced handgrip force and dexterity. Forklift drivers worked long hours (5 h/day) at below-zero temperatures and suffered cold symptoms even though they stay in the cold short times at one time [33]. As also shown here, the forklift drivers moved repeatedly between very cold and less cold working sites, which is known to cause thermal stress [32], and they are exposed to whole-body vibration [42], motion sickness [43], and carbon monoxide emissions [44], which may contribute to the reporting of symptoms. To our knowledge, previous studies on cold workplaces have not compared the prevalence of cold-related respiratory or circulatory symptoms, or performance degradation, among forklift drivers with those of workers in other job categories. One study (33) that compared handgrip force between forklift drivers working at very low (from − 20 °C to − 23 °C) and cool (12–15 °C) temperatures found no difference between these groups.

Cold-related respiratory symptoms, performance degradation, and thirst were also overrepresented among the storage and manufacturing workers as well as drying of the mouth among the storage workers. This finding could be attributed to the storage workers staying a relatively long time (3.8 h/day) at below-zero temperatures and the manufacturing workers staying a long time at 0–16 °C (6.9 h/day). A previous Thai study [12] compared cold storage and office workers in the frozen food industry and found a higher prevalence of respiratory symptoms among the cold storage workers but did not investigate performance degradation, thirst, and drying of the mouth. We are not aware of other studies comparing the latter symptoms between job categories, although the high prevalence of musculoskeletal, circulatory, and respiratory symptoms among cold workplace workers has been well documented [4, 8, 9, 27, 45]. While thirst and drying of the mouth suggest insufficient fluid intake and dehydration, they have not been previously described among cold workplace workers. Thus storage and manufacturing workers should be advised to keep hydrated.

Determining the significance of the reported cold-related symptoms among the forklift drivers and the storage and manufacturing workers is complicated, in that, workers at the coldest sites protected themselves better than those working at less cold sites. The prevalence of cold-related symptoms and complaints among these groups was still substantial and clearly indicates inadequate measures being taken against the cold.

Work-related issues due to the cold are reportedly more common among women than among men [5, 12]. We found a higher prevalence of cold-related cardiac and circulatory problems in women. This may partly reflect women’s greater propensity to perceive cold discomfort [46] and health-related issues in general [47]. The lower degree of performance degradation in women than in men could be attributed to differing physical requirements of work for women compared with men. In cold work, women may also work at a higher level of muscular activity [48], which produces heat and helps counteract cold-related performance degradation.

Cold related problems in concentration, motivation and endurance showed a curved age trend, an initial rise being followed by a decline in the highest age group - a pattern similar to that seen in the total number various cold-related symptoms. One likely underlying reason is health-based selection due to older workers shifting to lighter jobs or away from work. Especially the prevalence of cold-related thirst was low among the oldest workers. This could be attributed to the age-related decrease in the sense of thirst [49], which together with cold-induced diuresis and voluntary reduction of fluid intake, may lead to significant dehydration [50]. This trend was also shown in relation to cold-related dryness of the mouth, which could be related to age-related changes in oral dryness [51]. Thus, older workers are at risk of dehydration with consequent haemoconcentration, increased blood viscosity, and the risk of cardiovascular events [17]. Given this, measures should be put in place to ensure that older workers are informed of the need to keep hydrated rather than wait until they become thirsty.

Cold-related performance degradation in terms of motivation, endurance, handgrip force and dexterity was marginally less common among the obese workers than among those of normal weight. One explanation for this finding, not reported in other relevant studies [5, 12], could be the greater lean body mass among those who are obese, which increases heat production, while the insulation provided by a thicker fat layer is offset by greater heat loss from a larger body surface area [52]. Thus, workers of normal weight are likely to be more sensitive to the cold than those who are obese and would benefit from more protective clothing. The higher prevalence of cardiac symptoms and performance degradation among the ex-smokers can be explained in terms of likely deteriorating health and undiagnosed diseases due to their previous smoking history.

The finding of performance degradation related to workplace cold among frequent alcohol consumers was unsurprising but has not been previously described and clearly pinpoints an important area for more effective measures to maintain work ability. It was unclear why the frequent alcohol consumers reported less cold-related thirst than others. One possibility is that the blunted feeling of thirst under cold exposure [50] would be further suppressed through consuming alcohol, which causes anti-diuresis after initial diuresis and could thereby counteract dehydration and the feeling of thirst. Alternatively, some mechanisms in the dipsogenic centre in the brain unrelated to diuresis could play a role [53].

Less cold-related performance degradation, especially in terms of endurance and handgrip force, was reported among workers with a long employment history. This finding may reflect adaptation to the cold during a long employment time [54] or the elimination of symptomatic individuals from cold workplace work over time.

### Strengths and limitations

The strength of this study was that the prevalence figures were compared in terms of adjusted prevalence and prevalence differences. Compared with the customary practice of reporting odds ratios (OR) from logistic regression [5, 12], the use of adjusted prevalence and prevalence differences has certain advantages. First, it avoids the issue of ORs greatly over-emphasizing group-wise differences when symptoms are common, as was the case here. Second, the prevalence differences give the effect measures on an absolute scale and are more useful in evaluating the symptom burden among the population concerned. In this study, many prevalences were high (up to 90%) and calculating the relative differences between the groups would have underestimated the magnitude of preventive potential in the vulnerable groups. Third, a comparison of crude and adjusted prevalence figures also better illustrates the effect of confounding on group-wise prevalence patterns [25, 26].

Due to the convenience sampling method, caution should be exercised when interpreting the results. However, marked bias in the group-wise prevalence patterns was unlikely because they were carefully controlled for confounding factors. Although the sample was considered adequate for detecting even small or moderate prevalence differences, the estimates may still have been less accurate in some strata, which may not have allowed the detection of all potential differences between the groups. Because the symptoms are subjective perceptions, their validity cannot be assessed against any external gold standard. However, our previous experience [10, 15, 21, 22] points to adequate face validity. Additionally, the temperature of the products handled or that of the tools used was not measured in this study, which may have caused some bias [9]. Moreover, answers to the symptom questions may have been affected by specific cultural or socio-cultural factors that could not be controlled. Finally, the low number of workers with a diagnosed disease did not allow adjusting for pre-existing medical conditions.

## Conclusions

The findings of this study suggest that the current level of cold protection in this industry, especially among vulnerable groups, may not be sufficient to prevent the occurrence of cold-related symptoms. These symptoms not only cause suffering and worsen productivity but may also increase the likelihood of severe disease events in the long term. It is also important to be aware of which groups of workers are at risk of impaired physical and mental performance because adequate physical and mental functioning is critical for the safety of workers and his/her co-workers. Our findings demonstrate a need for more specific studies to clarify the adequacy of cold protection among cold workplace workers and the role of adaptation to a hot climate as a factor in cold sensitivity. This information is particularly relevant in light of global warming, which is likely to have a significant effect in this area [55] and thus increase the contrast between workplace cold and outdoor heat. Further studies are also needed to clarify whether cold workplace workers in a tropical climate are more vulnerable to the cold than their counterparts in cooler climates.

## Supplementary information

**Additional file 1: Table S1.** Numbers of workers and temperature in the base population and the sample studied, classified by factory sections. **Table S2.** Air temperature, relative humidity and wind velocity measured in each worker’s working space, classified by factory section, job category and education. N is the number of workers in each group. **Table S3.** Daily hours spent at temperatures < 0 °C and 0–16 °C and percentages of workers moving between cold and warm sites ≥4 times/day, classified by job category and education. N is the number workers in each group. **Table S4.** Temperature regarded as cold, classified by personal and work-related factors. **Table S5.** The mean number of individual cold-related symptoms, classified by personal and work-related factors.

**Additional file 2: Figure S1.** Prevalence of cold-related symptoms and complaints (%) by sex. Horizontal bars indicate 95% confidence intervals. M: males, F: females. **Figure S2.** Prevalence of cold-related symptoms and complaints (%) by age groups (years). Horizontal bars indicate 95% confidence intervals. **Figure S3.** Prevalence of cold-related symptoms and complaints (%) by educational classes. Horizontal bars indicate 95% confidence intervals. Univ: university, Voc: vocational school, High: high school, Mid: middle school, Prim: primary school. **Figure S4.** Prevalence of cold-related symptoms and complaints (%) by body mass index. Horizontal bars indicate 95% confidence intervals. Obe: obese (BMI ≥ 25.0 kg/m^2^), Over: overweight (BMI 23.0–24.9 kg/m^2^), Norm: normal weight (BMI ≤ 22.9 kg/m^2^). **Figure S5.** Prevalence of cold-related symptoms and complaints (%) by smoking. Horizontal bars indicate 95% confidence intervals. Smok: smoker, Ex: ex-smoker, Nev: never smoked. **Figure S6.** Prevalence of cold-related symptoms and complaints (%) by alcohol consumption. Horizontal bars indicate 95% confidence intervals. We: once a week or more often, Mon: monthly, Occ: occasionally, No: does not use alcohol. **Figure S7.** Prevalence of cold-related symptoms and complaints (%) by job categories. Horizontal bars indicate 95% confidence intervals. Fork: forklift driver, Stor: storage worker, Manu: manufacturing worker, Offi: office staff. **Figure S8.** Prevalence of cold-related symptoms and complaints (%) by employment years. Horizontal bars indicate 95% confidence intervals.

## Data Availability

The data are confidential and cannot be shared.

## Abbreviations

P: Prevalence
PD: Prevalence difference
pp.: Percentage point
CI: Confidence interval
