# Supplementary material for: Cold-related symptoms and performance degradation among Thai poultry industry workers with reference to vulnerable groups: a cross-sectional study
Source: BMC Public Health. 2020 Sep 4;20:1357. doi: 10.1186/s12889-020-09272-6 (PMC7487455; doi:10.1186/s12889-020-09272-6)
Supplement: Supplementary file 1 — Additional file 1: Table S1. Numbers of workers and temperature in the base population and the sample studied, classified by factory sections. Table S2. Air temperature, relative humidity and wind velocity measured in each worker’s working space, classified by factory section, job category and education. N is the number of workers in each group. Table S3. Daily hours spent at temperatures < 0 °C and 0–16 °C and percentages of workers moving between cold and warm sites ≥4 times/day, classified by job category and education. N is the number workers in each group. Table S4. Temperature regarded as cold, classified by personal and work-related factors. Table S5. The mean number of individual cold-related symptoms, classified by personal and work-related factors. [file 12889_2020_9272_MOESM1_ESM.docx]

| **Table S1** Numbers of workers and temperature in the base population and the sample studied, classified by factory sections | | | | | | | |
| --- | --- | --- | --- | --- | --- | --- | --- |
|  | Factory | | | | | | ALL |
|  | A | | | B | C | D |  |
| BASE POPULATION |  | | |  |  |  |  |
| No. of workers | 288 | | | 5034 | 500 | 7250 | 13,072 |
| Outdoor temperature °C (range) at 12 pm‒18 pm | | 31‒33 | | 31‒32 | 30‒34 | 28‒32 | 28‒34 |
| Indoor temperature °C (range) |  | | |  |  |  |  |
| Cold storage | -25‒4 | | | -20‒4 | -24‒4 | -35‒4 | -35‒4 |
| Production hall | 11‒14 | | | 9‒10 | -1‒14 | 12 | -1‒14 |
| Office | 23‒27 | | | 25 | 23‒27 | 25 | 23‒27 |
| SAMPLE INTERVIEWED |  | | |  |  |  |  |
| No. of workers | 59 | | | 145 | 70 | 148 | 422 |
| Cold storage | 5 | | | 44 | 32 | 85 | 166 |
| Production hall | 38 | | | 51 | 18 | 45 | 152 |
| Office | 14 | | | 47 | 16 | 18 | 95 |
| Other | 2 | | | 0 | 4 | 0 | 6 |
| SAMPLE WITH MEASUREMENTS | | |  |  |  |  |  |
| No. of workers | 42 | | | 78 | 68 | 116 | 304 |
| Cold storage | 5 | | | 35 | 31 | 71 | 142 |
| Production hall | 37 | | | 38 | 18 | 45 | 138 |
| Office | 0 | | | 4 | 15 | 0 | 19 |
| Other | 0 | | | 0 | 4 | 0 | 4 |
| Temperature °C (mean, range) | 5 (-20‒13) | | | 3 (-22‒14) | 12 (-19‒23) | -1 (-20‒2) | 4 (-22‒23) |
| Cold storage | -14 (-20‒0) | | | -7 (-22‒14) | 7 (-19‒23) | -3 (-20‒2) | -2 (-22‒23) |
| Production hall | 7 (0‒13) | | | 10 (10‒14) | 7 (-1‒14) | 2 (2‒2) | 6 (-1‒14) |
| Office | - | | | 14 (14‒14) | 23 (23‒23) | - | 21 (14‒23) |
| Other | - | | | - | 23 (23‒23) | - | 23 (23‒23) |
| The interviews were conducted on 13‒14 November 2017 (Factory A), on 22‒24 November 2017 (Factory B), on 11 July, 11 August, 11 September and 16 November 2017 (Factory C), and 27‒30 November 2017 (Factory D).  In factory B, three workers had missing information on factory section in the interviewed sample and one worker in the measurement sample. | | | | | | | |

| **Table S2** Air temperature, relative humidity and wind velocity measured in each worker’s working space, classified by factory section, job category and education. N is the number of workers in each group. | | | | | | | | |
| --- | --- | --- | --- | --- | --- | --- | --- | --- |
|  | Temperature | |  | Relative humidity | |  | Wind velocity | |
|  | °C  mean (range) | N |  | %  (range) | N |  | m/s  mean (range) | N |
| Factory section |  |  |  |  |  |  |  |  |
| Cold storage | -2 (-22‒23) | 142 |  | 41 (29‒72) | 139 |  | 0.37 (0.01‒2.20) | 135 |
| Production hall | 6 (-1‒14) | 138 |  | 49 (27‒72) | 138 |  | 0.51 (0.05‒3.00) | 138 |
| Office | 21 (14‒23) | 19 |  | 65 (63‒72) | 19 |  | 0.36 (0.05‒0.44) | 19 |
| Other | 23 (23‒23) | 4 |  | 63 (63‒63) | 4 |  | 0.44 (0.44‒0.44) | 4 |
| All sections | 4 (-22‒23) | 303 |  | 47 (27‒72) | 300 |  | 0.43 (0.01‒3.00) | 296 |
| Job category |  |  |  |  |  |  |  |  |
| Forklift driver | -20 (-22‒ -19) | 33 |  | 32 (29‒38) | 31 |  | 0.57 (0.22‒2.20) | 31 |
| Storage worker | 2 (-21‒14) | 94 |  | 41 (29‒72) | 93 |  | 0.30 (0.01‒2.20) | 89 |
| Manufacturing worker | 6 (-1‒14) | 141 |  | 50 (27‒72) | 141 |  | 0.50 (0.05‒3.00) | 141 |
| Office staff | 22 (14‒23) | 29 |  | 64 (63‒72) | 29 |  | 0.41 (0.05‒0.44) | 29 |
| All job categories | 4 (-22‒23) | 297 |  | 47 (27‒72) | 294 |  | 0.43 (0.01‒3.00) | 290 |
| Education |  |  |  |  |  |  |  |  |
| Primary school | 5 (-21‒14) | 64 |  | 48 (27‒72) | 64 |  | 0.44 (0.08‒3.00) | 64 |
| Middle school | 1 (-22‒23) | 92 |  | 45 (29‒72) | 90 |  | 0.38 (0.01‒1.25) | 89 |
| High school | -2 (-22‒14) | 63 |  | 41 (29‒68) | 62 |  | 0.47 (0.06‒2.20) | 62 |
| Vocational school | 8 (-20‒23) | 40 |  | 50 (32‒72) | 40 |  | 0.48 (0.05‒1.25) | 40 |
| University | 11 (-21‒23) | 42 |  | 55 (29‒72) | 42 |  | 0.42 (0.05‒2.20) | 39 |
| All educational classes | 4 (-22‒23) | 301 |  | 47 (27‒72) | 298 |  | 0.43 (0.01‒3.00) | 294 |
| Total | 4 (-22‒23) | 304 |  | 47 (27‒72) | 301 |  | 0.43 (0.01‒3.00) | 297 |
| The measurements of air temperature and relative humidity were conducted by using the Thermo-hygrometer 303 C (Shenzhen Graigar Technology, China), and air velocity was measured by the VelociCalc® 9545 (TSI Incorporated, Minnesota USA). The measurements were done at several points in each working area. In areas where temperature and air velocity varied, their minimum and maximum were recorded and expressed as averages. Relative humidity was stable in each area and was expressed as a single value. The values recorded in each working area were applied to the study participants in the same area. | | | | | | | | |

| **Table S3** Daily hours spent at temperatures < 0 ºC and 0–16 ºC and percentages of workers moving between cold and warm sites ≥ 4 times/day, classified by job category and education. N is the number workers in each group. | | | | | | | | |
| --- | --- | --- | --- | --- | --- | --- | --- | --- |
|  | Daily hours spent at < 0 ºC ^a^ | |  | Daily hours spent  at 0–16 ºC ^b^ | |  | Moving between  cold and warm sites  ≥ 4 times/day ^c^ | |
|  | hours | N |  | hours | N |  | % | N |
| Job category |  |  |  |  |  |  |  |  |
| Forklift driver | 5.1 | 33 |  | 0.9 | 33 |  | 87.9 | 33 |
| Storage worker | 3.8 | 95 |  | 2.7 | 95 |  | 74.7 | 95 |
| Manufacturing worker | 0.6 | 142 |  | 6.9 | 142 |  | 87.2 | 141 |
| Office staff | 0.2 | 134 |  | 0.6 | 134 |  | 70.1 | 134 |
| All | 1.5 | 404 |  | 3.3 | 404 |  | 77.2 | 403 |
| Education |  |  |  |  |  |  |  |  |
| University | 0.6 | 106 |  | 1.7 | 106 |  | 67.0 | 106 |
| Vocational school | 1.3 | 72 |  | 1.7 | 72 |  | 77.5 | 71 |
| High school | 2.4 | 72 |  | 3.4 | 72 |  | 87.5 | 72 |
| Middle school | 2.2 | 97 |  | 4.5 | 97 |  | 77.3 | 97 |
| Primary school | 1.5 | 69 |  | 5.7 | 69 |  | 82.6 | 69 |
| All | 1.5 | 416 |  | 3.3 | 416 |  | 77.2 | 415 |
| ^a^ Based on question: “How long do you work during one day at temperatures below 0 ºC (hours)” | | | | | | | | |
| ^b^ Based on question: “How long do you work during one day at temperatures 0–16 ºC (hours)” | | | | | | | | |
| ^c^ Based on question: “Do you move between cold and warm working spaces on most days?” (seldom or not at all / often (4 ̶ 6 times) / all the time (more than 6 times) ) | | | | | | | | |

| **Table S4** Temperature regarded as cold^a^, classified by personal and work-related factors. | | | |
| --- | --- | --- | --- |
| Characteristic | Coding | Mode  (ºC)^b^ | Workers regarding temperature   < 20 ºC as cold  % (no. of respondents) |
| Sex | Males | 20 | 48.5 (196) |
|  | Females | 20 | 45.1 (224) |
| Age (yr) | 18–24 | 20 | 49.0 (100) |
|  | 25–34 | 20 | 45.9 (135) |
|  | 35–44 | 20 | 43.3 (120) |
|  | 45–57 | 20 | 50.0 (64) |
| Education | Primary school | 20 | 34.8 (69) |
|  | Middle school | 20 | 35.0 (97) |
|  | High school | 20 | 41.7 (72) |
|  | Vocational school | 20 | 52.8 (72) |
|  | University | 20 | 63.5 (104) |
| Body mass index kg/m^2 c^ | Normal (≤ 22.9) | 20 | 53.7 (190) |
|  | Overweight (23.0–24.9) | 15 | 32.4 (68) |
|  | Obese (≥ 25.0) | 20 | 44.1 (161) |
| Smoking | Never smoked | 20 | 46.9 (305) |
|  | Ex-smoker | 20 | 64.7 (17) |
|  | Regular smoker | 20 | 42.9 (98) |
| Alcohol consumption | Does not consume alcohol | 20 | 41.7 (206) |
|  | Occasionally | 20 | 52.5 (101) |
|  | Monthly | 20 | 51.6 (62) |
|  | Weekly | 20 | 50.0 (48) |
| Job category | Forklift driver | 20 | 54.5 (33) |
|  | Storage worker | 20 | 53.7 (95) |
|  | Manufacturing worker | 10 | 16.2 (142) |
|  | Office staff | 20 | 70.5 (132) |
| Employment years | 0–1.9 | 20 | 51.1 (139) |
|  | 2–9.9 | 20 | 41.9 (136) |
|  | 10 + | 20 | 46.5 (142) |
| All |  | 20 | 47.7 (420) |
| ^a^ Based on question: “What temperature do you regard as cold (˚C) ?”  ^b^ The most common value of the distribution  ^c^ Classified according to World Health Organization [19] | | | |

| **Table S5** The mean number of individual cold-related symptoms, classified by personal and work-related factors. | | | |
| --- | --- | --- | --- |
| Characteristic | Coding | Mean number of symptoms (range) | |
| Sex | Males | 9.0 | (0–22) |
|  | Females | 10.1 | (0–22) |
| Age (yr) | 18–24 | 8.3 | (0–20) |
|  | 25–34 | 9.9 | (0–22) |
|  | 35–44 | 10.8 | (0–22) |
|  | 45–57 | 8.6 | (0–22) |
| Education | Primary school | 8.0 | (0–20) |
|  | Middle school | 8.8 | (0–22) |
|  | High school | 8.9 | (0–22) |
|  | Vocational school | 10.9 | (0–22) |
|  | University | 11.2 | (0–22) |
| Body mass index kg/m^2^ | Normal (≤ 22.9) | 9.7 | (0–22) |
|  | Overweight (23.0–24.9) | 9.3 | (0–22) |
|  | Obese (≥ 25.0) | 9.6 | (0–22) |
| Smoking | Never smoked | 10.1 | (0–22) |
|  | Ex-smoker | 9.2 | (1–22) |
|  | Regular smoker | 8.0 | (0–21) |
| Alcohol consumption | Does not consume alcohol | 9.7 | (0–22) |
|  | Occasionally | 9.6 | (0–22) |
|  | Monthly | 9.7 | (0–22) |
|  | Weekly | 9.2 | (0–18) |
| Job category | Forklift driver | 9.0 | (0–21) |
|  | Storage worker | 8.9 | (0–22) |
|  | Manufacturing worker | 9.4 | (0–22) |
|  | Office staff | 10.7 | (0–22) |
| Employment years | 0–1.9 | 9.0 | (0–22) |
|  | 2–9.9 | 9.7 | (0–22) |
|  | 10 + | 10.1 | (0–22) |
| All |  | 9.6 | (0–22) |
