# Supplementary material for: Cold-related symptoms and performance degradation among Thai poultry industry workers with reference to vulnerable groups: a cross-sectional study
Source: BMC Public Health. 2020 Sep 4;20:1357. doi: 10.1186/s12889-020-09272-6 (PMC7487455; doi:10.1186/s12889-020-09272-6)
Supplement: Supplementary file 2 — Additional file 2: Figure S1. Prevalence of cold-related symptoms and complaints (%) by sex. Horizontal bars indicate 95% confidence intervals. M: males, F: females. Figure S2. Prevalence of cold-related symptoms and complaints (%) by age groups (years). Horizontal bars indicate 95% confidence intervals. Figure S3. Prevalence of cold-related symptoms and complaints (%) by educational classes. Horizontal bars indicate 95% confidence intervals. Univ: university, Voc: vocational school, High: high school, Mid: middle school, Prim: primary school. Figure S4. Prevalence of cold-related symptoms and complaints (%) by body mass index. Horizontal bars indicate 95% confidence intervals. Obe: obese (BMI ≥ 25.0 kg/m2), Over: overweight (BMI 23.0–24.9 kg/m2), Norm: normal weight (BMI ≤ 22.9 kg/m2). Figure S5. Prevalence of cold-related symptoms and complaints (%) by smoking. Horizontal bars indicate 95% confidence intervals. Smok: smoker, Ex: ex-smoker, Nev: never smoked. Figure S6. Prevalence of cold-related symptoms and complaints (%) by alcohol consumption. Horizontal bars indicate 95% confidence intervals. We: once a week or more often, Mon: monthly, Occ: occasionally, No: does not use alcohol. Figure S7. Prevalence of cold-related symptoms and complaints (%) by job categories. Horizontal bars indicate 95% confidence intervals. Fork: forklift driver, Stor: storage worker, Manu: manufacturing worker, Offi: office staff. Figure S8. Prevalence of cold-related symptoms and complaints (%) by employment years. Horizontal bars indicate 95% confidence intervals. [file 12889_2020_9272_MOESM2_ESM.docx]

| **Explanations to Figures S1–S8** | |
| --- | --- |
| Symptom groups  (in capitals) | Individual symptoms |
| RESPIRATORY | At least one of the following symptoms (not shown separately in Figures): shortness of breath, mucus excretion, prolonged cough or wheezing |
| CARDIAC | At least one the following symptoms (not shown separately in Figures): chest pain or cardiac arrhythmia |
| CIRCULATION | At least one of the following symptoms (not shown separately in Figures): peripheral circulation disturbances, blurring of vision or migraine |
| FINGERS | At least one of the following symptoms (not shown separately in Figures): cold sensitive, white or blue fingers |
| GENERAL | At least one of the following symptoms (shown in lower case in Figures): sleep disturbances or intermittent sleep, unusually strong fatigue, thirst or dryness of mouth (shown in lower case in Figures) |
| PERFORMANCE | At least one of the following symptoms (shown in lower case in Figures): concentration, motivation, endurance, ability to hold, hand grip force or finger dexterity |

| \|  \| \| --- \| \| **** \|   **Figure S1** Prevalence of cold-related symptoms and complaints (%) by sex. Horizontal bars indicate 95% confidence intervals. M: males, F: females. |
| --- | --- | --- |

|  |
| --- |
| **F****igure S2** Prevalence of cold-related symptoms and complaints (%) by age groups (years). Horizontal bars indicate 95% confidence intervals. |

|  |
| --- |
| **Figure S3** Prevalence of cold-related symptoms and complaints (%) by educational classes. Horizontal bars indicate 95% confidence intervals. Univ: university, Voc: vocational school, High: high school, Mid: middle school, Prim: primary school. |

|  |
| --- |
| **Figure S4** Prevalence of cold-related symptoms and complaints (%) by body mass index. Horizontal bars indicate 95% confidence intervals. Obe: obese (BMI ≥ 25.0 kg/m^2^), Over: overweight (BMI 23.0–24.9 kg/m^2^), Norm: normal weight (BMI ≤ 22.9 kg/m^2^). |

|  |
| --- |
| **Figure S5** Prevalence of cold-related symptoms and complaints (%) by smoking. Horizontal bars indicate 95% confidence intervals. Smok: smoker, Ex: ex-smoker, Nev: never smoked. |

|  |
| --- |
| **Figure S6** Prevalence of cold-related symptoms and complaints (%) by alcohol consumption. Horizontal bars indicate 95% confidence intervals. We: once a week or more often, Mon: monthly, Occ: occasionally, No: does not use alcohol. |

|  |
| --- |
| **Figure S7** Prevalence of cold-related symptoms and complaints (%) by job categories. Horizontal bars indicate 95% confidence intervals. Fork: forklift driver, Stor: storage worker, Manu: manufacturing worker, Offi: office staff. |

|  |
| --- |
| **Figure S8** Prevalence of cold-related symptoms and complaints (%) by employment years. Horizontal bars indicate 95% confidence intervals. |
